# Cholinesterase inhibitor use in amyloid PET-negative mild cognitive impairment and cognitive changes

**DOI:** 10.1186/s13195-024-01580-y

**Published:** 2024-10-02

**Authors:** Jung-Min Pyun, Young Ho Park, Min Ju Kang, SangYun Kim

**Affiliations:** 1 Department of Neurology, Soonchunhyang University Seoul Hospital, Soonchunhyang University College of Medicine, 59, Daesagwan-ro, Yongsan-gu, Seoul, 04401 Republic of Korea; 2 Department of Neurology, Seoul National University Bundang Hospital, Seoul National University College of Medicine, 82, Gumi-ro 173 Beon-gil, Bundang-gu, Seongnam-si, 13620 Gyeonggi-do Republic of Korea; 3 Department of Neurology, Veterans Health Service Medical Center, 53, Jinhwangdo-ro 61-gil, Gangdong-gu, Seoul, 05368 Republic of Korea

**Keywords:** Cholinesterase inhibitors, Mild cognitive impairment, Alzheimer’s disease, Cognitive decline

## Abstract

**Background:**

Cholinesterase inhibitors (ChEIs) are prescribed for Alzheimer’s disease (AD) and sometimes for mild cognitive impairment (MCI) without knowing underlying pathologies and its effect on cognition. We investigated the frequency of ChEI prescriptions in amyloid-negative MCI and their association with cognitive changes in the Alzheimer’s Disease Neuroimaging Initiative (ADNI) cohort.

**Methods:**

We included participants with amyloid positron emission tomography (PET)-negative MCI from the ADNI. We analyzed the associations of ChEI use with cognitive changes, brain volume, and cerebrospinal fluid (CSF) total tau (t-tau), hyperphosphorylated tau_181_ (p-tau_181_), and p-tau_181_/t-tau ratio.

**Results:**

ChEIs were prescribed in 27.4% of amyloid PET-negative MCI and were associated with faster cognitive decline, reduced baseline hippocampal volume and entorhinal cortical thickness, and a longitudinal decrease in the frontal lobe cortical thickness.

**Conclusions:**

The association between ChEI use and accelerated cognitive decline may stem from underlying pathologies involving reduced hippocampal volume, entorhinal cortical thickness and faster frontal lobe atrophy. We suggest that ChEI use in amyloid PET-negative MCI patients might need further consideration, and studies investigating the causality between ChEI use and cognitive decline are warranted in the future.

**Supplementary Information:**

The online version contains supplementary material available at 10.1186/s13195-024-01580-y.

## Background

 The cholinergic hypothesis suggests the loss of cholinergic neurons in the basal forebrain as a pathomechanism of Alzheimer’s disease (AD), leading to cognitive impairment [1]. Cholinesterase inhibitors (ChEIs) decrease the breakdown of acetylcholine at the synapse, enhancing cholinergic signaling [2]. ChEIs, including donepezil, rivastigmine, and galantamine, are widely used for symptomatic treatment of AD.

The cognitive effects of ChEIs have been observed in Alzheimer’s disease (AD) and other neurodegenerative dementias, such as Parkinson’s disease (PD) dementia and dementia with Lewy bodies [3–5]. In AD, ChEIs can reduce amyloid-β (Aβ) levels and increase cholinergic activity. In PD, where cholinergic activity is diminished due to early α-synuclein accumulation in the cholinergic neurons of the basal forebrain, ChEIs have demonstrated beneficial effects [6, 7]. Furthermore, ChEIs may counteract neuroinflammation and oxidative stress in neurodegenerative diseases [5]. Although studies have indicated potential cognitive benefits of ChEIs in non-AD cognitive impairment, the evidence remains insufficient [8]. 

Another issue in the clinical practice is the use of ChEIs in individuals with mild cognitive impairment (MCI). The practice guideline suggested by Petersen et al. recommended against offering ChEIs in MCI due to the lack of level A evidence [9]. Despite the lack of robust evidence, a previous study with the Alzheimer’s Disease Neuroimaging Initiative (ADNI) cohort reported that 44% of the recruited patients with MCI were treated with ChEIs [10]. Published in 2011, although potential discrepancies from current practice due to the time difference might exist, the finding that 44% of patients were treated with ChEIs is substantial. The lack of clear evidence for a beneficial effect of ChEIs on cognition might be due to the heterogeneous underlying pathologies in MCI and the unavailability of biomarkers confirming these pathologies. Physicians have had to assume underlying causes of MCI based on clinical manifestations. The recent development of biomarkers allows for the confirmation of amyloidopathy in MCI, which can be used to select appropriate targets of ChEIs. Therefore, understanding the effect of ChEIs on cognition in individuals with amyloid-negative MCI might provide helpful clues for developing treatment strategies.

This study aimed to investigate the frequency of ChEI prescription in patients with amyloid PET-negative MCI and the association of ChEI use with longitudinal cognitive changes. Additionally, we compared baseline and longitudinal changes in brain cortical thickness and hippocampal volume on magnetic resonance imaging (MRI). Moreover, the study analyzed total tau (t-tau), hyperphosphorylated tau_181_ (p-tau_181_), and p-tau_181_/t-tau ratio in cerebrospinal fluid (CSF) between ChEI users and non-users to explore underlying potential tauopathy.

## Methods

### Participants

**Fig. 1:**
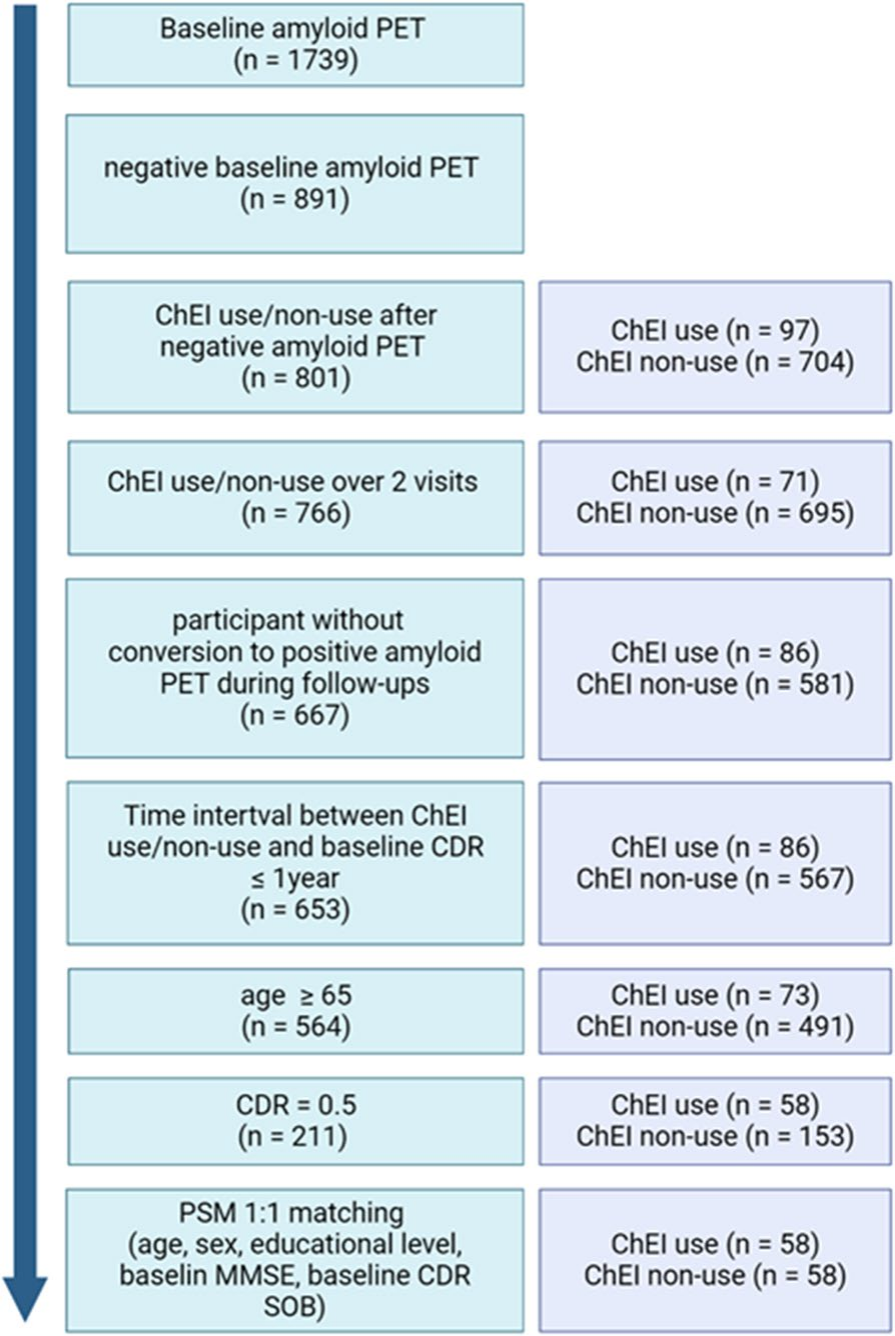
Schematic describing the selection process of study participants. Abbreviation: CDR, clinical dementia rating; CDR SOB, clinical dementia rating sum of boxes; ChEI, cholinesterase inhibitor; MMSE, mini-mental state examination; PET, positron emission tomography; PSM, propensity score matching

We used data from the ADNI database (http://adni.loni.usc.edu). ADNI launched in 2003 as a public–private partnership, primarily aims to test whether neuroimaging, other biological markers, and clinical neuropsychological assessment can be combined to measure the progression of MCI and early AD. For this study, we aimed to include patients with MCI who had negative amyloid PET scans. The process of participant selection is depicted in Fig. 1. Initially, participants with negative baseline amyloid PET scans were included (*n* = 891). Among them, 801 participants were confirmed to be using or not using ChEIs (donepezil, rivastigmine, or galantamine) after undergoing amyloid PET. Subsequently, participants with consistent ChEI use or non-use during at least two visits were selected (*n* = 766). From these 766 participants, those who did not convert to positive amyloid PET scans during follow-ups were chosen (*n* = 667). Furthermore, participants with a time interval between ChEI use or non-use and baseline clinical dementia rating (CDR) score of less than one year or the same were included (*n*= 653) [11]. Among them, participants aged 65 years or older were selected (*n* = 564). Finally, participants with MCI with a CDR score of 0.5 were included (*n* = 211).

### Cognitive assessment

Cognitive function was evaluated using Mini-Mental State Exam (MMSE), CDR, CDR Sum of Boxes (CDR SOB), and composite scores of memory (ADNI MEM) and executive function (ADNI EF) [12–14]. 

### Amyloid PET

Amyloid PET scans were performed using [18 F] florbetaben (FBB) or [18 F] florbetapir (FBP). The cutoff for determining amyloid positivity or negativity was based on centiloid (CL) value. We defined amyloid PET negativity as a CL value lesser than 19 [15, 16]. Of the total 1,739 baseline amyloid PET scans, 1,360 scans (78.2%) used FBP, while 379 scans (21.8%) used FBB. The distribution of amyloid PET tracers for each group in unmatched and matched cohorts are shown in Supplementary Table 1. Additional information on amyloid PET processing can be found online (https://adni.loni.usc.edu/methods/documents/).

### Brain MRI

Structural brain volume was estimated from T1-weighted brain MRI scans using FreeSurfer (surfer.nmr.mgh.harvard.edu) [17]. Details on brain MRI processing are available online (https://adni.loni.usc.edu/methods/documents/). For this study, we used hippocampal volume and cortical thickness for each frontal, parietal, temporal, occipital lobes, cingulate cortex, entorhinal cortex, and parahippocampal cortex [18]. Hippocampal volume was calculated as the average value of right and left hippocampal volumes divided by the estimated intracranial volume (ICV) and expressed as a percentage ((mm3/ICV) ×100). Cortical thickness for the frontal lobe was determined as the average value of right and left frontal pole, precentral, superior frontal, caudal middle frontal, rostral middle frontal, and medial orbitofrontal cortical thicknesses; cortical thickness for the parietal lobe, as the average value of right and left postcentral, superior parietal, supramarginal, paracentral, and inferior parietal cortical thicknesses; cortical thickness for the temporal lobe, as the average value of right and left temporal pole, fusiform, superior temporal, inferior temporal, and middle temporal cortical thicknesses; cortical thickness for the occipital lobe was used the average value of right and left lingual, lateral occipital, pericalcarine, and cuneus cortical thicknesses; and cortical thickness for cingulate cortex, as the average value of right and left posterior cingulate, rostral anterior cingulate, caudal anterior cingulate, and isthmus cingulate cortical thicknesses. We calculated entorhinal and parahippocampal cortical thicknesses as the average values of the left and right cortical thicknesses for these regions. Baseline MRI was defined as the MRI scan performed at the baseline ChEI use or non-use time and within one year.

### CSF measurement

The concentrations of CSF p-tau_181_ and t-tau were measured using the micro-bead-based multiplex immunoassay, the INNO-BIA AlzBio3 RUO test (Fujirebio, Ghent, Belgium) on the Luminex platform [19]. Qualification of the analytical performance of CSF samples from ADNI was controlled, showing a within-center coefficient of variation (%CV) 95% confidence interval (CI) value (mean) of 6.4–6.8% (6.7%) for t-tau, and 5.5–18.0% (10.8%) for p-tau_181_ [20]. The inter-center %CV 95% CI ranged from 9.6 to 15.2% (13.1%) for t-tau, and 11.3–18.2% (14.6%) for p-tau_181_. Detailed information on data processing can be found online (https://adni.loni.usc.edu/methods/documents/). Baseline CSF was defined as CSF performed at the baseline ChEI use or non-use time and within one year.

### Statistical analysis

To minimize treatment selection bias and differences in baseline characteristics between participants using ChEI and those not using it, a propensity score matching (PSM) analysis was conducted. Propensity scores were calculated through logistic regression with covariates of baseline age, educational level, CDR SOB, and MMSE using the Matchit packages in R. Participants using ChEI and those not using it were matched in a 1:1 ratio based on these propensity scores, with a caliper size of 0.2. We used the propensity score-matched cohort for all further analyses.

Association analyses between ChEI use and longitudinal cognition were performed using a linear mixed model adjusted for age, sex, educational levels, and baseline entorhinal cortical thickness. Association of ChEI with baseline and longitudinal MRI and CSF measurements were analyzed using linear regression and linear mixed models. Age, sex, and MRI field strength were adjusted in MRI analyses, and age and sex were adjusted in CSF analyses. We included longitudinal data of cognitive assessments, MRI, and CSF measurements with follow-up times of up to 60 months due to a substantial decrease in the frequency of measurements. The total observation number of longitudinal measurements and the mean number of assessments per individual are listed in Supplementary Table 2.

We used the R software (version 4.2.3) for all analyses, with statistical significance set at *p* < 0.05. Multiple comparisons were not performed and the p-values are nominal.

## Results

### Frequency of chei use in amyloid PET-negative MCI

Before PSM matching, 211 participants were included. Among these, 58 patients (27.4%) with amyloid PET-negative MCI were prescribed ChEI (Table 1). Participants using ChEI were older, with lower MMSE scores and higher CDR SOB scores than those not using ChEI. After PSM matching, the imbalances in age, baseline MMSE score, and baseline CDR SOB score were alleviated. Of the 58 amyloid-negative MCI patients using ChEIs, 45 patients (77.5%) were prescribed donepezil, 8 patients (13.7%) received rivastigmine, and 5 patients (8.6%) were prescribed galantamine.

**Table 1:** Demographics and clinical characteristics of participants before and after propensity score matching

	Before matching		*p*-value	After matching		*p*-value
	ChEI use (*n* = 58)	ChEI non-use (*n* = 153)		ChEI use (*n* = 58)	ChEI non-use (*n* = 58)	
Age, years	76.4 ± 6.4	74.4 ± 6.6	0.047	76.4 ± 6.4	76.8 ± 7.1	0.786
Female	22 (37.9)	65 (42.4)	0.658	22 (37.9)	17 (29.3)	0.432
Education, years	16.5 ± 2.5	16.3 ± 2.5	0.673	16.5 ± 2.5	16.7 ± 2.4	0.654
Baseline MMSE	27.5 ± 2.1	28.1 ± 1.7	0.036	27.5 ± 2.1	27.7 ± 1.7	0.570
Baseline CDR SOB	1.6 ± 0.9	1.3 ± 0.8	0.024	1.6 ± 0.9	1.5 ± 0.9	0.658
Baseline ADNI MEM	0.1 ± 0.6	0.6 ± 0.7	< 0.001	0.1 ± 0.6	0.4 ± 0.6	0.012
Baseline ADNI EF	0.2 ± 0.6	0.5 ± 0.8	0.023	0.2 ± 0.6	0.2 ± 0.8	0.929
*APOEε*4 carrier	5 (9.2)	19 (13.5)	0.566	5 (9.2)	8 (15.0)	0.530
Amyloid PET, Centiloid	-3.8 ± 13.1	-2.9 ± 9.5	0.635	-3.8 ± 13.1	-1.4 ± 9.7	0.265
Baseline brain MRI	*n* = 54	*n* = 130		*n* = 54	*n* = 45	
Hippocampal volume, (mm^3^/ICV) ×100	0.2 ± 0.0	0.2 ± 0.0	< 0.001	0.2 ± 0.0	0.2 ± 0.0	0.065
Cortical thickness, mm						
Frontal lobe	2.3 ± 0.1	2.3 ± 0.1	0.455	2.3 ± 0.1	2.3 ± 0.1	0.219
Parietal lobe	2.1 ± 0.1	2.1 ± 0.1	0.323	2.1 ± 0.1	2.1 ± 0.1	0.532
Temporal lobe	2.7 ± 0.2	2.8 ± 0.2	0.016	2.7 ± 0.2	2.7 ± 0.2	0.787
Occipital lobe	1.81 ± 0.1	1.8 ± 0.1	0.855	1.8 ± 0.1	1.8 ± 0.1	0.711
Cingulate cortex	2.5 ± 0.1	2.5 ± 0.1	0.565	2.5 ± 0.1	2.5 ± 0.1	0.189
Entorhinal cortex	3.0 ± 0.5	3.4 ± 0.4	< 0.001	3.0 ± 0.5	3.3 ± 0.4	0.028
Parahippocampal cortex	2.5 ± 0.3	2.6 ± 0.3	0.153	2.5 ± 0.3	2.6 ± 0.3	0.720
Baseline CSF	*n* = 44	*n* = 103		*n* = 44	*n* = 33	
p-tau_181_, pg/mL	26.3 ± 10.8	24.3 ± 13.4	0.387	26.3 ± 10.8	26.8 ± 16.5	0.879
t-tau, pg/mL	79.1 ± 65.9	89.9 ± 75.5	0.411	79.1 ± 65.9	101.8 ± 89.8	0.204
p-tau_181_/t-tau ratio	0.4 ± 0.2	0.4 ± 0.2	0.219	0.4 ± 0.2	0.4 ± 0.3	0.736

Data are presented as the mean ± standard deviation or n (%)

*Abbreviation*: *ADNI EF *Alzheimer’s Disease Neuroimaging Initiative composite score of executive function, *ADNI MEM *Alzheimer’s Disease Neuroimaging Initiative composite score of memory, *CDR SOB *Clinical dementia rating sum of boxes, *ChEI *Cholinesterase inhibitor, *CSF *Cerebrospinal fluid, *ICV* Estimated intracranial volume, *MMSE *Mini-mental state examination, *MRI *Magnetic resonance imaging, *PET *Positron emission tomography; p-tau_181_, hyperphosphorylated tau_181;_ t-tau, total tau

### Ch use and longitudinal cognitive changes

**Fig. 2:**
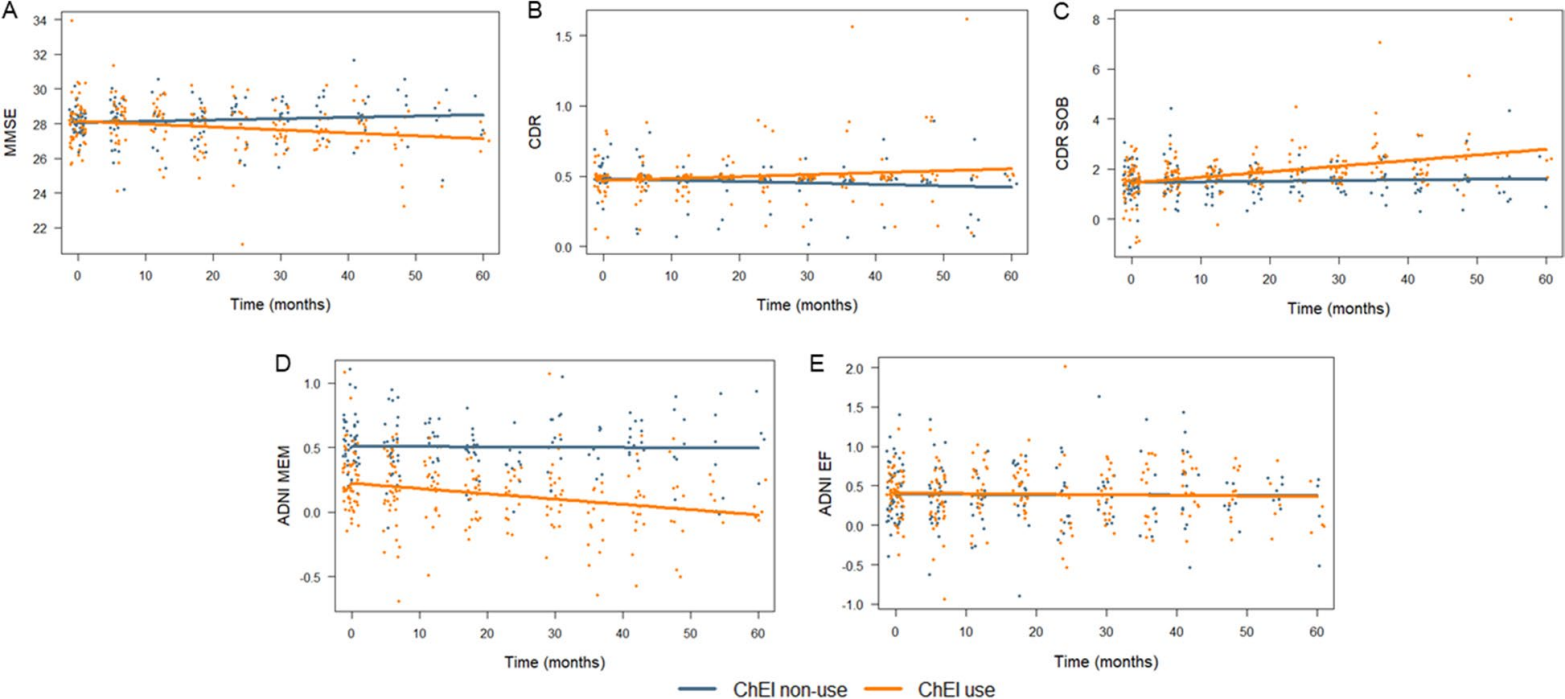
Association of ChEI use with longitudinal cognitive changes. The association between ChEI use/non-use and longitudinal cognitive function was depicted as residual plots for MMSE (A), CDR (B), CDR SOB (C), ADNI MEM (D), and ADNI EF (E). Abbreviation: ADNI EF, Alzheimer’s Disease Neuroimaging Initiative composite score of executive function; ADNI MEM, Alzheimer’s Disease Neuroimaging Initiative composite score of memory; CDR, clinical dementia rating; CDR SOB, clinical dementia rating sum of boxes; ChEI, cholinesterase inhibitors; MMSE mini-mental state examination

Associations between ChEI use and longitudinal cognitive function assessed by MMSE, CDR, CDR SOB, ADNI MEM, and ADNI EF, were analyzed. ChEI use was significantly associated with accelerated cognitive decline in MMSE (β = -2.44 × 10 − 3, *p* = 0.009), CDR (β = 2.40 × 10 − 3, *p* = 0.019), CDR SOB (β = 1.98 × 10 − 2, *p* < 0.001), and ADNI MEM (β = -3.85 × 10 − 3, *p* = 0.023) (Table 2). ADNI EF was unrelated to ChEI use (β = -4.11 × 10 − 4, *p* = 0.876). The cognitive changes depending on ChEI use are shown in Fig. 2.

**Table 2:** Association between ChEI use and longitudinal cognitive changes

	β	*p*-value
MMSE	-2.44$\:\times\:$10^−3^	0.009
CDR	2.40$\:\times\:$10^−3^	0.019
CDR SOB	1.98$\:\times\:$10^−2^	< 0.001
ADNI MEM	-3.85$\:\times\:$10^−3^	0.023
ADNI EF	-4.11$\:\times\:$10^−4^	0.876

The models were adjusted for age, sex, educational levels, and baseline entorhinal cortex thickness on MRI. Linear mixed model was performed and the interaction terms between ChEI use and time were assessed

*Abbreviation*: *ADNI EF *Alzheimer’s Disease Neuroimaging Initiative composite score of executive function, *ADNI MEM *Alzheimer’s Disease Neuroimaging Initiative composite score of memory, *CDR *Clinical dementia rating, *CDR SOB *Clinical dementia rating sum of boxes, *ChEI *Cholinesterase inhibitors, *MMSE *Mini-mental state examination, *MRI *Magnetic resonance imaging

### ChEI use and brain MRI

Before matching, the ChEI use group showed decreased baseline hippocampal volume and temporal and entorhinal cortical thicknesses compared to the ChEI non-use group (Table 1). After matching, the ChEI use group still showed decreased entorhinal cortical thickness compared to the ChEI non-use group (3.0 ± 0.5 vs. 3.3 ± 0.4, *p* = 0.028). In linear regression analyses adjusted for age, sex, and MRI field strength, ChEI use was associated with reduced baseline hippocampal volume (β = -1.67 × 10 − 4, *p* = 0.037) and entorhinal cortical thickness (β = -2.20 × 10 − 1, *p* = 0.035) (Table 3). ChEI use was not related to baseline cortical thicknesses of the frontal, parietal, temporal, occipital lobes, cingulate cortex, and parahippocampal cortex. In the linear mixed model, ChEI use was significantly associated with decreased frontal lobe cortical thickness (β = -3.51 × 10 − 5, *p* = 0.027). There was no significant association between ChEI use and longitudinal hippocampal volume, parietal, temporal, occipital lobes, cingulate, and parahippocampus.

**Table 3:** Association of ChEI use with baseline and longitudinal hippocampal volume and cortical thicknesses

	Baseline (β, *p*-value)	Longitudinal (β, *p*-value)
Hippocampal volume	-1.67$\:\times\:$10^−4^, 0.037	-3.47$\:\times\:$10^−9^, 0.858
Cortical thickness		
Frontal lobe	3.78$\:\times\:$10^−3^, 0.219	-3.51$\:\times\:$10^−5^, 0.027
Parietal lobe	1.63$\:\times\:$10^−2^, 0.555	-2.35$\:\times\:$10^−5^, 0.104
Temporal lobe	3.94$\:\times\:$10^−3^, 0.926	-1.61$\:\times\:$10^−5^, 0.363
Occipital lobe	8.55$\:\times\:$10^−3^, 0.712	-3.31$\:\times\:$10^−6^, 0.760
Cingulate cortex	3.67$\:\times\:$10^−2^, 0.278	-1.85$\:\times\:$10^−5^, 0.193
Entorhinal cortex	-2.20$\:\times\:$10^−1^, 0.035	2.27$\:\times\:$10^−5^, 0.547
Parahippocampal cortex	-9.15$\:\times\:$10^−3^, 0.889	1.31$\:\times\:$10^−5^, 0.535

The models were adjusted for age, sex, and MRI field strength. Cross-sectional analyses with baseline MRI images were performed using linear regression model. Longitudinal analyses were performed using linear mixed model and the interaction terms between ChEI use and time were assessed

*Abbreviation*: *ChEI *Cholinesterase inhibitors, *MRI *Magnetic resonance imaging

### ChEI use and CSF measurements

In both unmatched and matched cohorts, there was no significant difference in CSF p-tau_181_, t-tau levels, and p-tau_181_/t-tau ratio between ChEI use and ChEI non-use groups (Table 1). The linear regression model adjusted for age and sex showed that ChEI use was not associated with the baseline levels of p-tau_181_ (β = -2.89 × 10 − 1, *p* = 0.928), t-tau (β = -2.42 × 101, *p* = 0.182), and p-tau_181_/t-tau ratio (β = 3.56 × 10 − 2, *p* = 0.610) (Table 4). Moreover, ChEI use was not related to longitudinal changes in p-tau_181_ (β = 9.29 × 10 − 4, *p* = 0.767) and t-tau (β = 6.13 × 10 − 3, *p* = 0.833) levels and p-tau_181_/t-tau ratio (β = 1.82 × 10 − 5, *p* = 0.821).

**Table 4:** Association of ChEI use with baseline and longitudinal level of CSF p-tau_181_, t-tau, and p-tau_181_/t-tau ratio

	Baseline (β, *p*-value)	Longitudinal (β, *p*-value)
p-tau_181_	-2.89$\:\times\:$10^−1^, 0.928	9.29$\:\times\:$10^−4^, 0.767
t-tau	-2.42$\:\times\:$10^1^, 0.182	6.13$\:\times\:$10^−3^, 0.833
p-tau_181_/t-tau ratio	3.56$\:\times\:$10^−2^, 0.610	1.82$\:\times\:$10^−5^, 0.821

The models were adjusted for age and sex were adjusted. Cross-sectional analyses with baseline CSF measurements were performed using a linear regression model. Longitudinal analyses were performed using a linear mixed model and the interaction terms between ChEI use and time were assessed

*Abbreviation*: *ChEI *Cholinesterase inhibitors, *CSF *Cerebrospinal fluid; p-tau_181_, hyperphosphorylated tau_181;_ t-tau, total tau

## Discussion

This study found that 27.4% of participants with amyloid PET-negative MCI were prescribed ChEIs. ChEI use was associated with faster cognitive decline as measured by MMSE, CDR, CDR SOB, and ADNI MEM. The ChEI use group was also related to reduced baseline hippocampal volume, entorhinal cortical thickness, and a faster longitudinal decrease in frontal lobe cortical thickness. In CSF analysis, ChEI use was not associated with baseline and longitudinal levels of p-tau_181_, t-tau, and p-tau_181_/t-tau ratio.

The frequency of ChEI prescriptions after undergoing amyloid PET was 27.4%. Before PSM, participants using ChEI tended to be older, with poorer cognitive function, and showed reduced hippocampal volume, temporal lobe, and entorhinal cortical thicknesses. These factors may influence ChEI prescription due to their association with disease severity [21–23]. 

We used age, sex, educational level, MMSE score, and CDR SOB as covariates for PSM to minimize differences in baseline cognitive function. After matching, the ChEI use group showed poor baseline ADNI MEM score and reduced entorhinal cortical thickness. In the matched cohort, ChEI use was associated with faster cognitive decline, adjusted for age, sex, educational levels, and baseline entorhinal cortical thickness. Specifically, ChEI was related to a faster decline in MMSE, CDR, and CDR SOB, although the three cognitive scores were similar between groups at baseline. ChEI use was associated with a faster decline in ADNI MEM but not ADNI EF. This finding suggests that individuals using ChEI may have pathologies that predominantly affect memory function, such as limbic-predominant age-related TAR DNA-binding protein 43 (TDP-43) encephalopathy (LATE) [24]. LATE has been reported to cause predominant memory impairment and hippocampal atrophy, showing similarities in clinical phenotype to AD. However, the effect of baseline ADNI MEM differences between groups needs consideration while interpreting ADNI MEM results. When ADNI MEM was additionally included as a covariate in the PSM, the significant association between faster decline in MMSE, CDR, CDR-SOB, and ADNI MEM scores with ChEI use remained unchanged (Supplementary Tables 3 and 4). Moreover, although we limited the follow-up period to 60 months due to a substantial decrease in the frequency of measurements, we also provided analyses without this limitation, extending up to 126 months (Supplementary Table 5), as cognitive assessments over more than 10 years are a strength of the ADNI data. The statistical significance of the results remained unchanged.

In brain MRI analysis, ChEI use was related to reduced baseline hippocampal volume and entorhinal cortical thickness in linear regression analysis. Hippocampal atrophy and entorhinal thinning are observed in early AD, but also in other neurodegenerative diseases, such as LATE, hippocampal sclerosis, and frontotemporal dementia (FTD) [25]. Baseline brain atrophy might affect ChEI prescription as a poor prognostic factor along with poor cognitive function [22, 23]. Longitudinally, ChEI use was significantly associated with a faster decrease in frontal lobe cortical thickness. This finding indicates that individuals in the ChEI use group may harbor pathologies related to faster frontal lobe atrophy, such as frontotemporal lobe degeneration (FTLD). A previous study has reported several anatomical subtypes of behavioral variant FTD. The frontal dominant subtype presented frontal lobe atrophy and was related to a faster CDR SOB increase [26]. Moreover, behavioral variant FTD has been demonstrated with episodic memory impairment, and memory dysfunction was one of the key features of disease progression, consistent with our findings [27]. 

Next, we compared CSF tau levels between groups to explore tau pathological profiles, which might contribute to differences in cognitive progression and brain atrophy. ChEI use was not associated with baseline and longitudinal p-tau_181_, t-tau, and p-tau_181_/t-tau ratio. Increased p-tau_181_ could provide a clue for primary age-related tauopathy or reduced p-tau_181_/t-tau ratio would suggest FTLD-TDP, distinguishing from FTLD-Tau [28, 29]. No significant difference in CSF tau profile between ChEI use and non-use groups in our study could be due to a small sample size, missing values, short follow-up period, or heterogeneous underlying pathologies.

In this study, amyloid PET negativity was defined using a threshold CL value of less than 19. However, patients with subthreshold CL values between 12 and 18, indicating moderate levels of amyloidopathy, could influence the results [30]. In the matched cohort, 8 participants (5 ChEI users and 3 ChEI non-users) had CL values between 12 and 18. We excluded these 8 patients and reanalyzed the data to account for the effect of moderate levels of amyloidopathy. The statistical significance of the results remained unchanged when including patients with CL < 12. Therefore, we demonstrated that the potential impact of subthreshold amyloidopathy did not affect the overall results.

We investigated the concordance of amyloid negativity in PET and CSF in our study population. Baseline CSF Aβ_42_ data were available for 147 and 77 patients in the unmatched and matched cohorts, respectively. Amyloid negativity was defined as CSF Aβ_42_> 192 pg/mL [31]. Among the 147 amyloid PET-negative MCI patients in the unmatched cohort, 130 were amyloid-negative based on CSF Aβ_42_ levels, yielding a concordance of 88.4%. In the matched cohort, 69 out of 77 patients (89.6%) showed amyloid-negative CSF results. These rates were comparable to the concordance reported in another cohort [32]. 

Additionally, we compared baseline 2-[18 F]fluoro-2-deoxy-d-glucose (FDG) PET and AV1451 PET data between the ChEI user and non-user groups in the matched cohort to assess glucose metabolism and tau burden. There was no difference in tau uptake in the entorhinal region or in the composite regions of interest (ROI), which included the bilateral median uptake in the entorhinal, amygdala, parahippocampal, fusiform, inferior temporal, and middle temporal regions. Similarly, no difference in FDG uptake was observed in the composite ROI, which comprised the right and left angular gyri, bilateral posterior cingulate gyrus, and left middle/inferior temporal gyrus, between the groups (Supplementary Table 6). However, the available tau and FDG PET data were limited, and a larger sample size is needed for statistical significance.

### Limitations

This study has several limitations. First, the sample size was small, warranting validation in a large cohort. Second, as an association analysis, it cannot establish causality, requiring cautious interpretation of the results. Third, despite efforts to minimize selection bias through PSM, residual bias may remain. Fourth, the lack of biomarkers to identify the pathological characteristics of the groups is a limitation.

Nonetheless, this study revealed the frequency with which physicians treat amyloid PET-negative MCI with ChEIs and showed the association of ChEIs with faster cognitive decline. Although brain MRI analyses results showed the possible inclusion of frontal lobe related pathology, participants in our cohort may have various mixed pathologies of neurodegeneration such as tauopathy, vasculopathy, and synucleinopathy. These findings underscore the urgent need for accessible biomarkers for mixed pathologies to refine medical treatment with ChEI.

## Conclusions

In summary, our study demonstrated that the frequency of ChEI prescription in participants with amyloid PET-negative MCI was 27.4% in the ADNI cohort. ChEI use was associated with faster cognitive decline. ChEI use was also related to reduced baseline hippocampal volume and entorhinal cortical thickness, and a longitudinal decrease in the frontal lobe cortical thickness. The association between ChEI use and accelerated cognitive decline may stem from underlying pathologies involving reduced hippocampal volume, entorhinal cortical thickness and faster frontal lobe atrophy. We suggest that ChEI use in amyloid PET-negative MCI patients might need further consideration, and studies investigating the causality between ChEI use and cognitive decline in neurodegenerative diseases are warranted in the future.

## Supplementary information

Supplementary Material 1

Supplementary Material 2

Supplementary Material 3

Supplementary Material 4

Supplementary Material 5

Supplementary Material 6

## Data Availability

All demographic, imaging, and fluid biomarker data in this study were publicly available and downloaded from the ADNI database (http://adni.info.org).

## Abbreviations

Aβ: Amyloid-β
AD: Alzheimer’s disease
ADNI EF: Alzheimer’s Disease Neuroimaging Initiative composite score of executive function
ADNI MEM: Alzheimer’s Disease Neuroimaging Initiative composite score of memory
CDR: Clinical dementia rating
CDR SOB: Clinical dementia rating sum of boxes
ChEI: Cholinesterase inhibitor
CI: Confidence interval
CSF: Cerebrospinal fluid
MCI: Mild cognitive impairment
MMSE: Mini mental state examination
MRI: Magnetic resonance image
PET: Positron emission tomography
PSM: Propensity score matching
SUVR: Standardized uptake value ratio
