# Supplementary material for: Cholinesterase inhibitor use in amyloid PET-negative mild cognitive impairment and cognitive changes
Source: Alzheimers Res Ther. 2024 Oct 2;16:210. doi: 10.1186/s13195-024-01580-y (PMC11448210; doi:10.1186/s13195-024-01580-y)
Supplement: Supplementary file 1 — Supplementary Material 1 [file 13195_2024_1580_MOESM1_ESM.docx]

Supplementary Table 1. The distribution of amyloid PET tracers for each group.

| PET tracer | Before matching | | | After matching | | |
| --- | --- | --- | --- | --- | --- | --- |
|  | ChEI use  (n = 58) | ChEI  non-use  (n = 153) | *p*-value | ChEI use  (n = 58) | ChEI  non-use  (n = 58) | *p*-value |
| FBB | 6 (10.3%) | 24 (15.6%) | 0.441 | 6 (10.3%) | 13 (22.4%) | 0.132 |
| FBP | 52 (89.6%) | 129 (84.3%) |  | 52 (89.6%) | 45 (77.5%) |  |

Abbreviation: ChEI, cholinesterase inhibitor; FBB, [18F] florbetaben; FBP, [18F] florbetapir; PET, positron emission tomography.
