# Supplementary material for: Cholinesterase inhibitor use in amyloid PET-negative mild cognitive impairment and cognitive changes
Source: Alzheimers Res Ther. 2024 Oct 2;16:210. doi: 10.1186/s13195-024-01580-y (PMC11448210; doi:10.1186/s13195-024-01580-y)
Supplement: Supplementary file 2 — Supplementary Material 2 [file 13195_2024_1580_MOESM2_ESM.docx]

Supplementary Table 2. Number of missing values and longitudinal observation in baseline and longitudinal MRI, CSF, and cognitive function measurements.

|  | Before matching | | After matching | |
| --- | --- | --- | --- | --- |
| Baseline | ChEI use  (n = 58) | ChEI non-use (n = 153) | ChEI use  (n = 58) | ChEI non-use  (n = 58) |
| MRI missing values | 4 | 23 | 4 | 13 |
| CSF missing values | 14 | 50 | 14 | 25 |
| Longitudinal | Mean number of assessments per individual | | Observational number | |
| MMSE/ CDR (n = 99) | 3.6 | | 388 | |
| ADNI MEM/ EF (n = 99) | 3.8 | | 400 | |
| MRI (n = 99) | 3.9 | | 389 | |
| CSF (n = 77) | 3.0 | | 240 | |

Abbreviation: ADNI EF, Alzheimer’s Disease Neuroimaging Initiative composite score of executive function; ADNI MEM, Alzheimer’s Disease Neuroimaging Initiative composite score of memory; CDR, clinical dementia rating; CDR, clinical dementia rating; ChEI, cholinesterase inhibitor; CSF, cerebrospinal fluid; MMSE, mini-mental state examination; MRI, magnetic resonance imaging.
