# Supplementary material for: Cholinesterase inhibitor use in amyloid PET-negative mild cognitive impairment and cognitive changes
Source: Alzheimers Res Ther. 2024 Oct 2;16:210. doi: 10.1186/s13195-024-01580-y (PMC11448210; doi:10.1186/s13195-024-01580-y)
Supplement: Supplementary file 3 — Supplementary Material 3 [file 13195_2024_1580_MOESM3_ESM.docx]

Supplementary Table 3. Demographics and clinical characteristics of participants before and after propensity score matching, including age, sex, educational level, MMSE, CDR SOB, and ADNI MEM as covariates.

|  | Before matching | | *p*-value | After matching | | *p*-value |
| --- | --- | --- | --- | --- | --- | --- |
|  | ChEI use  (n = 58) | ChEI  non-use  (n = 153) |  | ChEI use  (n = 58) | ChEI  non-use  (n = 58) |  |
| Age, years | 76.4 ± 6.4 | 74.4 ± 6.6 | 0.047 | 76.4 ± 6.4 | 76.8 ± 7.1 | 0.765 |
| Female | 22 (37.9) | 65 (42.4) | 0.658 | 22 (37.9) | 20 (34.4) | 0.847 |
| Education, years | 16.5 ± 2.5 | 16.3 ± 2.5 | 0.673 | 16.5 ± 2.5 | 16.7 ± 2.5 | 0.659 |
| Baseline MMSE | 27.5 ± 2.1 | 28.1 ± 1.7 | 0.036 | 27.5 ± 2.1 | 27.6 ± 1.7 | 0.813 |
| Baseline CDR SOB | 1.6 ± 0.9 | 1.3 ± 0.8 | 0.024 | 1.6 ± 0.9 | 1.6 ± 1.0 | 0.925 |
| Baseline ADNI MEM | 0.1 ± 0.6 | 0.6 ± 0.7 | < 0.001 | 0.1 ± 0.6 | 0.2 ± 0.5 | 0.433 |
| Baseline ADNI EF | 0.2 ± 0.6 | 0.5 ± 0.8 | 0.023 | 0.2 ± 0.6 | 0.2 ± 0.8 | 0.974 |
| *APOEε*4 carrier | 5 (9.2) | 19 (13.5) | 0.566 | 5 (9.2) | 11 (21.5) | 0.138 |

Data are presented as the mean ± standard deviation or n (%).

Abbreviation: ADNI EF, Alzheimer’s Disease Neuroimaging Initiative composite score of executive function; ADNI MEM, Alzheimer’s Disease Neuroimaging Initiative composite score of memory; CDR SOB, clinical dementia rating sum of boxes; ChEI, cholinesterase inhibitor; MMSE, mini-mental state examination.
