# Supplementary material for: Cholinesterase inhibitor use in amyloid PET-negative mild cognitive impairment and cognitive changes
Source: Alzheimers Res Ther. 2024 Oct 2;16:210. doi: 10.1186/s13195-024-01580-y (PMC11448210; doi:10.1186/s13195-024-01580-y)
Supplement: Supplementary file 5 — Supplementary Material 5 [file 13195_2024_1580_MOESM5_ESM.docx]

Supplementary Table 5. Association between ChEI use and longitudinal cognitive changes without follow-up period limitation.

|  | β | *p*-value |
| --- | --- | --- |
| MMSE | -1.60$\times$10^-2^ | 0.005 |
| CDR | 5.11$\times$10^-3^ | < 0.001 |
| CDR SOB | 3.63$\times$10^-2^ | < 0.001 |
| ADNI MEM | -4.75$\times$10^-3^ | < 0.001 |
| ADNI EF | -2.20$\times$10^-3^ | 0.145 |

The models were adjusted for age, sex, educational levels, and baseline entorhinal cortex thickness on MRI. Linear mixed model was performed and the interaction terms between ChEI use and time were assessed.

Abbreviation: ADNI EF, Alzheimer’s Disease Neuroimaging Initiative composite score of executive function; ADNI MEM, Alzheimer’s Disease Neuroimaging Initiative composite score of memory; CDR, clinical dementia rating; CDR SOB, clinical dementia rating sum of boxes; ChEI, cholinesterase inhibitors; MMSE, mini-mental state examination; MRI, magnetic resonance imaging.
