# Supplementary material for: Cholinesterase inhibitor use in amyloid PET-negative mild cognitive impairment and cognitive changes
Source: Alzheimers Res Ther. 2024 Oct 2;16:210. doi: 10.1186/s13195-024-01580-y (PMC11448210; doi:10.1186/s13195-024-01580-y)
Supplement: Supplementary file 6 — Supplementary Material 6 [file 13195_2024_1580_MOESM6_ESM.docx]

Supplementary Table 6. A comparison of baseline FDG PET and tau PET between ChEI use and ChEI non-use groups in the matched cohort.

|  | ChEI use | ChEI non-use | *p*-value |
| --- | --- | --- | --- |
| Baseline tau PET | n = 9 | n = 13 |  |
| Composite-ROI | 1.14 [1.10-1.22] | 1.17 [1.15-1.24] | 0.512 |
| Entorhinal | 1.14 [1.01-1.19] | 1.11 [1.06-1.26] | 0.647 |
| Baseline FDG PET | n = 44 | n = 44 |  |
| Composite-ROI | 1.2 ± 0.0 | 1.2 ± 0.0 | 0.358 |

Abbreviation: ChEI, cholinesterase inhibitor; FDG, 2-[18F]fluoro-2-deoxy-d-glucose; ROI, regions of interest; PET, positron emission tomography.

The values are presented as median [interquartile range] or mean ± standard deviation.
